# Duodenal Bulb Adenocarcinoma: A Case Report

**DOI:** 10.7759/cureus.79996

**Published:** 2025-03-03

**Authors:** Mohammad N Kloub, Abdul-Rahman I Abusalim, Abdelhadi Farouji, Mohamed Eldesouki, Atheer Anwar, Raed Atiyat, Mehul Shah

**Affiliations:** 1 Internal Medicine, Saint Michael's Medical Center, Newark, USA; 2 Internal Medicine, University of Wisconsin School of Medicine and Public Health, Madison, USA; 3 School of Medicine, Mutah University, Karak, JOR; 4 Gastroenterology and Hepatology, Saint Michael's Medical Center, Newark, USA

**Keywords:** adenocarcinoma, duodenal bulb, duodenal cancer, gastrointestinal cancer surgery, primary small bowel cancer

## Abstract

The duodenum is thought to be the most prevalent site of primary small bowel adenocarcinoma, and the disease mostly affects the descending part of the duodenum. Although the majority of small intestine adenocarcinomas are caused by duodenal adenocarcinomas, it is extremely uncommon for them to occur in the duodenal bulb. We report the case of an 80-year-old female with a complex medical history who presented with symptomatic anemia secondary to gastrointestinal (GI) blood loss secondary to adenocarcinoma of the duodenal bulb.

## Introduction

Duodenal adenocarcinomas account for approximately 0.3-0.5% of all gastrointestinal (GI) malignancies and are considered a rare form of GI cancer; it was first described by Hamburger in 1746 [1,2]. Although duodenal adenocarcinomas can develop at any location within the duodenum, they most commonly arise in the descending part of the duodenum (D2) [1,2]. Despite their overall rarity, primary malignant tumors of the duodenum are recognized as the most common malignancy of the small intestine [3]. This is of particular importance, as the incidence of small bowel malignancies has been increasing, with duodenal adenocarcinoma showing a particularly rapid rise among the various types of small bowel cancer [3].

Due to the nonspecific nature of symptoms associated with duodenal adenocarcinoma, the majority of patients are diagnosed in the advanced stages, resulting in a poor prognosis, high medical complexity, and elevated mortality rates [4]. In this report, we present a case of duodenal adenocarcinoma in an exceptionally rare site. Additionally, we engage in a concise review of the literature on duodenal adenocarcinoma, with a particular emphasis on its occurrence within the duodenal bulb.

This article was previously posted to the Semantic Scholar preprint server on May 25, 2024.

## Case presentation

An 80-year-old female with a past medical history of hypertension, dyslipidemia, and ischemic stroke with residual right-sided weakness, who was currently on aspirin, atorvastatin, and amlodipine, presented complaining of fatigue and generalized weakness for the past few weeks before presentation. These symptoms had been getting worse over time, limiting her ability to perform activities of daily living. According to the patient, she was also experiencing shortness of breath with palpitations on minimal exertion, along with decreased appetite. She denied any chest pain, fever, chills, cough, weight loss, night sweats, changes in urinary habits, vomiting, diarrhea, bloody stool, or melena.

Upon examination, vital signs were reassuring (heart rate: 88 beats per minute, respiratory rate: 16 breaths per minute, temperature: 36.8 °C, and SpO_2_: 95%). She was pale but not in any acute distress. The abdomen was soft, with minimal tenderness in the epigastric area; no organomegaly, guarding, or rigidity; and normal bowel sounds. The digital rectal exam was unremarkable. The chest exam showed good air entry bilaterally with no added sounds. The cardiac exam revealed normal S1, S2, and regular rate and rhythm, with no murmurs detected. The neurologic exam was stable from prior stroke with residual weakness in the right upper and lower limbs.

Blood test results (Table 1) were significant for microcytic anemia and elevated tumor markers (Table 2) including carbohydrate antigen (CA) 125, CA 19-9, and carcinoembryonic antigen (CEA). She also tested positive for stool occult blood. Otherwise, she had a normal comprehensive metabolic panel.

**Table 1:** Basic blood tests BUN: blood urea nitrogen; MCV: mean corpuscular volume; WBC: white blood cells

Parameter	Patient value	Reference range
Hemoglobin	6.3 g/dl	12-15.5 g/dl
MCV	79 fL	81.6-98.3 fL
WBC	10.9 10^3^/uL	4.40-11.0 10^3^/uL
Platelets	492 10^3^/uL	15-450 10^3^/uL
BUN	12 mg/dL	6.0-24.0 mg/dl
Creatinine	0.70 mg/dL	0.5-1.0 mg/dl

**Table 2:** Tumor markers CA 19-9: carbohydrate antigen 19-9; CEA: carcinoembryonic antigen

Parameter	Patient value	Reference range
CA 19-9	>700 U/ml	0-34 U/ml
CEA	8.3 ng/ml	0-3 ng/ml

**Figure 1:**
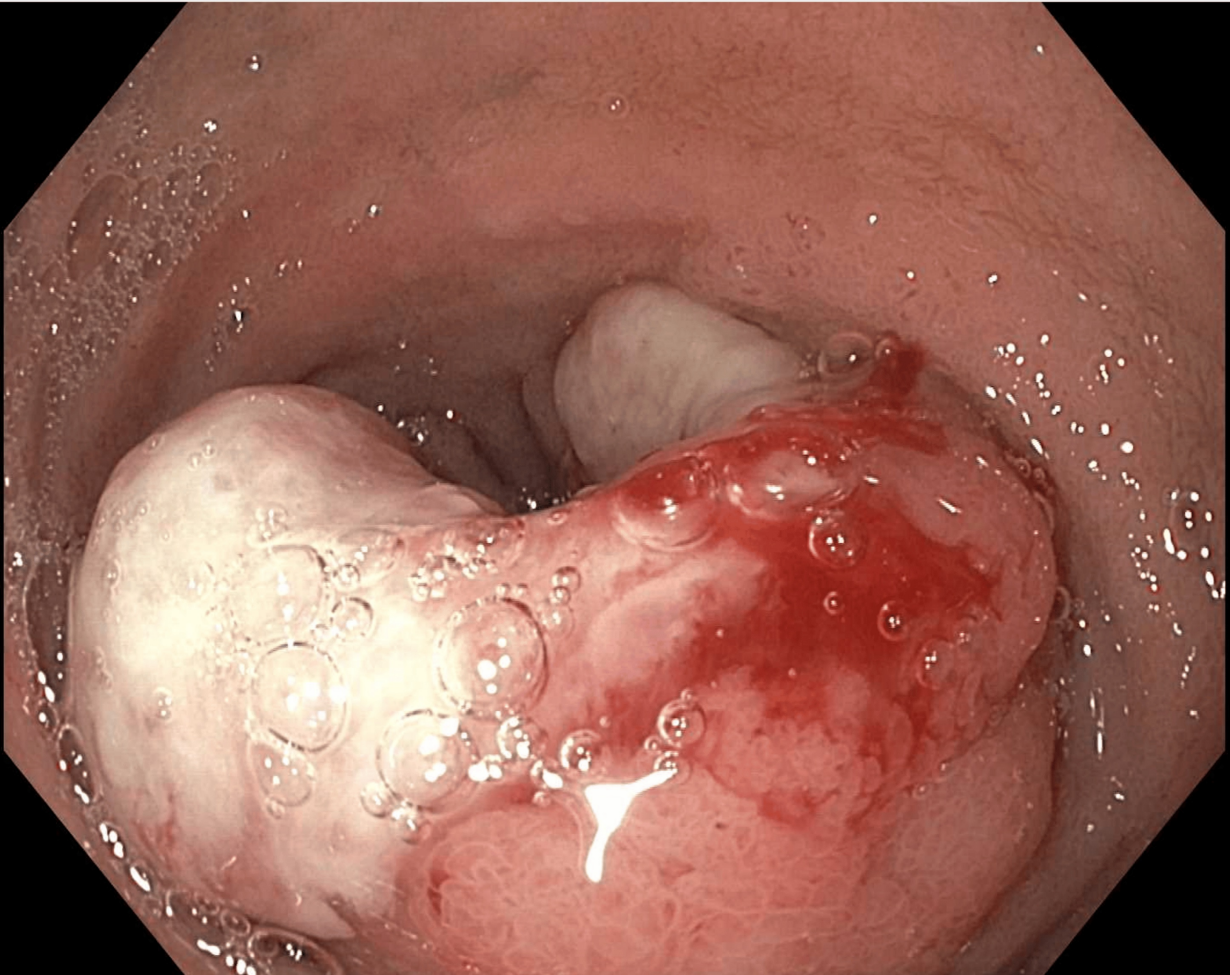
EGD image showing duodenal bulb large fungating, 5 cm infiltrating mass with partial obstruction of the lumen of the duodenal bulb extending to the second portion of the duodenum EGD: esophagogastroduodenoscopy

Esophagogastroduodenoscopy (EGD) revealed a distal duodenal bulb large fungating, 5 cm infiltrating mass with partial obstruction of the lumen of the duodenal bulb extending to the second portion of the duodenum (Figure 1). 

**Figure 2:**
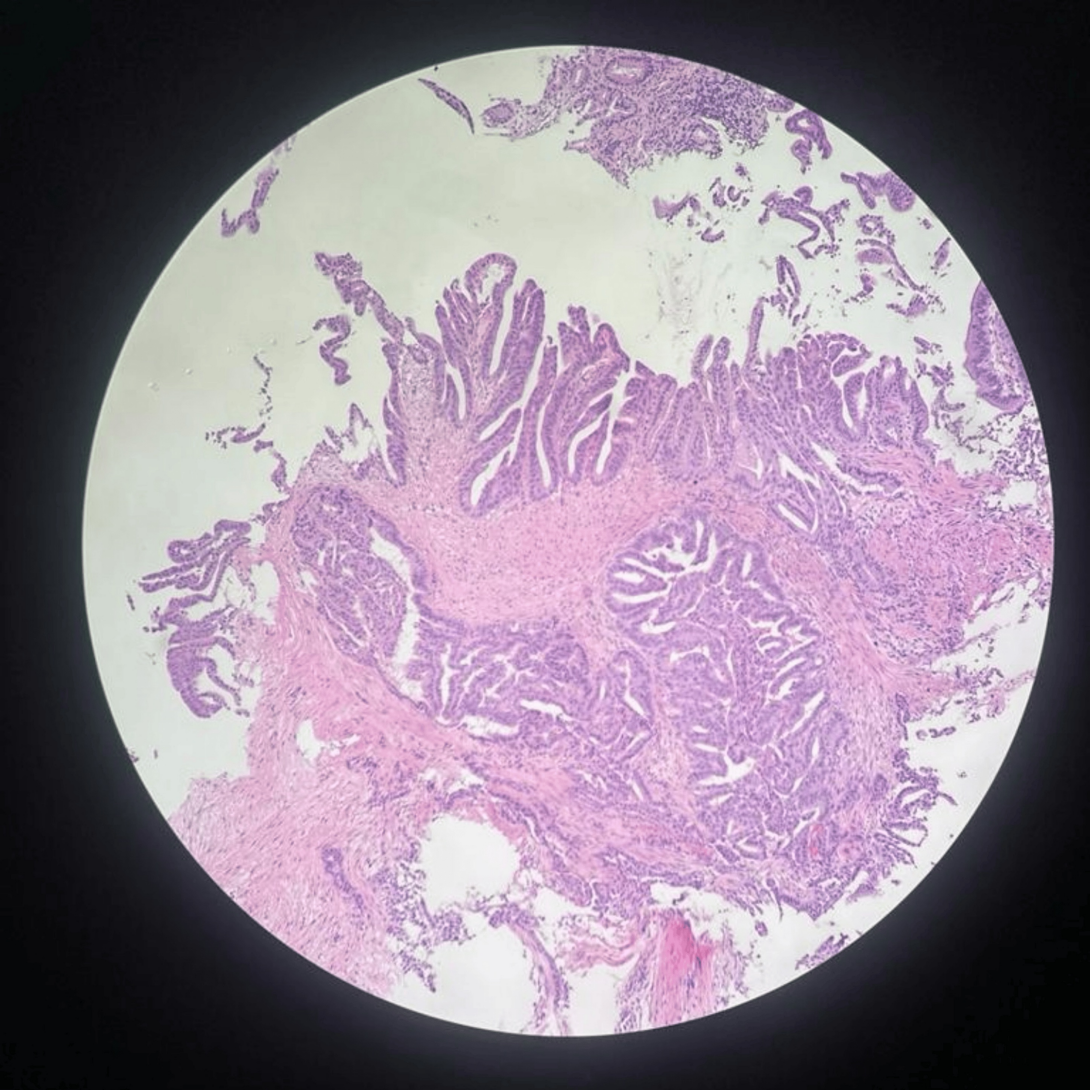
Histopathology slide of the duodenal bulb biopsy stained with H&E showing infiltrating adenocarcinoma, moderately differentiated H&E: hematoxylin and eosin

Biopsies from the lesion revealed infiltrating adenocarcinoma, moderately differentiated (Figure 2). Immunohistochemical stains (IHC) showed the tumor positive for cytokeratin 7 (CK7) and CK 19, and weakly positive for paired box gene 8 (PAX8). IHC was negative for cytokeratin 20 (CK 20), caudal-type homeobox transcription factor 2 (CDX2), and special AT-rich sequence-binding protein 2 (SATB2).

CT of the chest, abdomen, and pelvis performed after EGD showed an area of hypoattenuation in the region of the gastric antrum and proximal duodenum along with an adjacent enlarged lymph node measuring 1.4 x 1.2 cm, raising concerns for neoplastic involvement. The scan was negative for distant metastasis.

The patient remained in stable condition while the above workup was performed. She was transfused with two units of packed red blood cells (PRBCs) upon presentation for symptomatic treatment of her anemia, with stabilization of hemoglobin levels >8 on repeat testing and no acute drops in hemoglobin or need for further transfusions during her stay. Her fatigue, weakness, and shortness of breath improved significantly post-transfusion. She was discharged with a referral to the oncology and surgical services for interdisciplinary decision-making regarding further management.

## Discussion

The small intestine comprises the majority of the entire length of the GI tract. However, it is a rare location for cancer and accounts for less than 0.5% of all GI malignancies [1]. Adenocarcinoma is the most common malignant tumor of the GI tract, and the duodenum is considered the most common site for small intestine adenocarcinoma [1,2]. While duodenal adenocarcinoma most commonly occurs in the descending part of the duodenum, adenocarcinoma can originate from any part of the duodenum, including the duodenal bulb [5,6]. Patients usually present with advanced disease, and symptoms are usually vague and nonspecific, including abdominal pain, vomiting, intestinal obstruction, and weight loss, with overall survival after five years of 25-35% [2]. There are no apparent predisposing factors for duodenal cancer in the literature. However, duodenal adenomas, such as familial adenomatous polyposis, increase the risk. Alcohol, coffee, smoking, and dietary factors also seem to be risk factors [6]. Interestingly, our patient had none of these risk factors.

EGD is considered the first-line diagnostic modality for the evaluation of duodenal cancer, which allows for direct visualization and biopsy of lesions with a high diagnostic rate. In addition, endoscopic ultrasound (EUS) may be performed simultaneously to evaluate local extension or lymphadenopathy [6]. Several tumors with different histogenesis can develop in the GI tract. Frequently, it is difficult to distinguish between primary tumors and metastatic malignancies of unknown origin. Immunohistochemistry enables the use of various antibodies with the integrated evaluation of specific staining and can lead to a correct diagnosis [7]. Using cytokeratin, mucins, and catenin might be quite beneficial in most circumstances. In general, small intestinal adenocarcinomas, as in our case, have diffuse positivity for CK7 and lower CK20 and CDX2 expression rates than large intestine adenocarcinomas [7].

In the absence of metastatic disease, surgical resection with lesion-free margins is the mainstay of treatment for duodenal adenocarcinoma [2,6]. For adenocarcinoma of the first and second parts of the duodenum, pancreaticoduodenectomy is the recommended surgical technique [2,6]. In contrast, segmental resection is frequently reserved for adenocarcinoma of the third and fourth parts [6]. Pancreaticoduodenectomy is still considered a superior surgical intervention in terms of wider resection margin and more extensive regional lymph node clearance when compared with segmental resection [6]. Neoadjuvant chemotherapy has been implemented and shown to have positive results with better clinical outcomes in some reports, especially in the case of metastatic disease [8,9].

Duodenal bulb adenocarcinoma is exceptionally rare compared to malignancies in other parts of the duodenum. While the exact causes are yet to be discovered, it has been suggested that the duodenal bulb mucosa may be physiologically and immunologically privileged to escape oncogenic transformation [5].

## Conclusions

Duodenal adenocarcinoma is a rare GI malignancy, and it is associated with a high level of morbidity and mortality due to the late presentation of patients, usually with advanced disease. Duodenal adenocarcinoma most commonly occurs in the second part of the duodenum, and duodenal bulb adenocarcinoma is exceptionally rare. In this report, we highlight the importance of considering duodenal malignancies, even in unusual locations like the duodenal bulb, when evaluating patients with nonspecific GI symptoms, including upper GI bleeding. Early recognition, prompt endoscopic evaluation, and surgical intervention are crucial for the successful management of these patients.
